# A combined deformable model and medical transformer algorithm for medical image segmentation

**DOI:** 10.1007/s11517-022-02702-0

**Published:** 2022-11-03

**Authors:** Zhixian Tang, Jintao Duan, Yanming Sun, Yanan Zeng, Yile Zhang, Xufeng Yao

**Affiliations:** 1 College of Medical Imaging, Shanghai University of Medicine & Health Sciences, Shanghai, 201318 China; 2 Radiology Department, Shanghai University of Medicine & Health Sciences Affiliated Jiading Hospital, Shanghai, 201800 China; 3 College of Medical Instrumentation, Shanghai University of Medicine & Health Sciences, Shanghai, 201318 China

**Keywords:** Medical image segmentation, Image augmentation, Medical transformer, Deformable model

## Abstract

Deep learning–based segmentation models usually require substantial data, and the model usually suffers from poor generalization due to the lack of training data and inefficient network structure. We proposed to combine the deformable model and medical transformer neural network on the image segmentation task to alleviate the aforementioned problems. The proposed method first employs a statistical shape model to generate simulated contours of the target object, and then the thin plate spline is applied to create a realistic texture. Finally, a medical transformer network was constructed to segment three types of medical images, including prostate MR image, heart US image, and tongue color images. The segmentation accuracy of the three tasks achieved 89.97%, 91.90%, and 94.25%, respectively. The experimental results show that the proposed method improves medical image segmentation performance.

**Graphical abstract:**

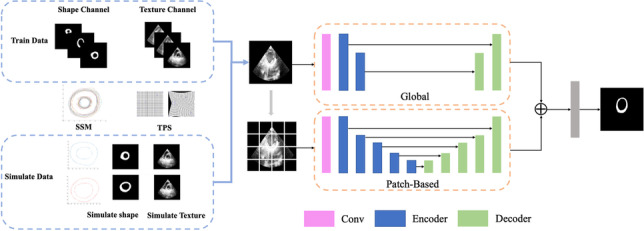

## Introduction

Imaging techniques have become essential for disease diagnosis, surgical planning, and prognostic evaluation in medical institutions [1]. Precisely segmenting the regions of interest (ROI) in these images can assist doctors in making a correct diagnosis of the disease. In clinical decision-making, image segmentation technology can provide a reliable basis for computer-aided diagnosis and treatment [2]. It is also critical for quantitative analysis [3] and surgical navigation [4]. Hence, image segmentation has important theoretical significance and clinical value.

Deep learning-based automatic segmentation algorithms have made significant progress [5]. Many deep learning methods have been successfully applied in cell segmentation [6], lung segmentation [7], prostate segmentation [8], brain structure segmentation [9], and fetal segmentation [10]. Training a robust segmentation model requires a large quantity of labeled data. However, physical professionals obtain the labeled data manually, which is time-consuming and laborious. Thus, the available annotated training data is limited. Moreover, the deep features of medical images are challenging to excavate. Therefore, many typical deep learning models do not perform well in medical image segmentation tasks. In addition, different structures and tuning strategies are usually required for various segmentation tasks to achieve the best for the respective tasks. Recently, lots of methods have been proposed to overcome the above difficulties, which can be roughly divided into the following categories:

The first category is to augment the training data. The most typical technology is rigid transformation, including rotation, translation, scaling, and tangent. Patch sampling [11] is also an effective data augmentation method. For example, Bertram et al. [12] used a content-sensitive sampling strategy for patchwise training. The emergence of Generative Adversarial Nets (GAN) provides a new idea for image augmentation. Huang [13] and Chong et al. [14] used GAN to synthesize brain images, improving subsequent image post-processing performance. Frid-Adar et al. [15] utilized GAN to generate some simulated images and improved the performance of CNN for liver lesion classification. However, most of these image data augmentation methods ignore the inherent properties of the image, and GAN tends to cause mode collapse.

The second category is to adopt the transfer learning strategy. Transfer learning can apply additional data or an existing model to a relevant task. For example, Dou's work [16] applied a transfer learning method for cardiac CT image segmentation using a pre-trained model on MR images. Martin et al. [17] proposed a 2D to 3D transfer learning method, the initial weights of the 3D Res-Unet were transferred from the 2D VGG-16. Transfer learning can speed up the convergence of the model for the second task and even improve its performance.

The third type is to integrate information from different layers or extract the long-range dependencies. The most classical network is the U-net proposed by Ronneberger et al. [18], which combines the image’s low-level and high-level convolutional features. Thus, it can achieve medical image segmentation with less training data. Recently, many U-net variants have been proposed. Representative networks include Attention U-net [19] for CT prostate segmentation, R2AU-net [20], U-Net +  + [21] and nn-Unet for multi-task segmentation of medical images. Some researchers try to change the convolution kernel's structure so that the image's multi-scale information can be utilized. For example, the Atrous convolution kernel [22] has a large receptive field, so each convolution output contains an extensive range of information. Wang et al. [23] proposed a method that addressed the gridding artifacts by smoothing the dilated convolution. Dai et al.[24]. used deformable convolution and deformable ROI pooling to enhance the transformation modeling capability of CNNs. Recently, Image GPT [25] can be perceived as a significant breakthrough in image processing whose success is mainly attributed to the self-attention mechanisms investigating the Transformer [26]. The Transformer can dig out the long-range dependencies. In the image segmentation tasks, the Transformer also performed well, such as MedT [27], Axial-Deeplab [28], TransU-net [29]. However, taking non-local attention as an example, the computational load is large, especially when the feature map is large and the computational efficiency is very low.

Inspired by the fundamental mechanism of the Transformer, we combined the deformable model and medical transformer network for medical image segmentation. First, we established a statistical shape model from the contours of the target object in the real training images. Then, we used the model to generate simulated contours of the target object. Second, we applied the thin plate spline to create a realistic texture. Third, we introduced the axial-attention and built a medical transformer network to segment three types of medical images, including prostate MR images, heart US images, and tongue images.

The contributions of this paper can be summarized as follows:

- We proposed an image augmentation strategy to alleviate the problem of data scarcity in medical image processing with deep neural networks.
- The network effectively applied axial attention and the dual-scale training strategy to mining the long-range feature information.
- We built the network and validated the model using three different types of data, including MRI images, ultrasound images, and color images.

The rest of this paper is organized as follows: Section 2 describes the framework of the method in detail, including image enhancement (Section 2.1), gated axial-attention mechanism (Section 2.2) and medical transformer (Section 2.3). We evaluate our method on three different datasets in Section 3 and discuss the advantages as well as disadvantages of the model in Section 4. We conclude the whole paper in Section 5.

## Methods

**Fig. 1:**
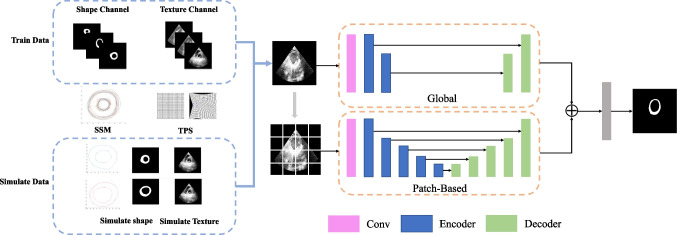
The framework of the proposed method

The proposed framework for medical image segmentation is shown in Fig. 1, containing the following steps.

- Image preprocessing. We employ intensity normalization and resample the original series to make the spatial resolution consistent in each direction.
- Image data augmentation. This step generates some simulated images with the deformable model and the improved thin plate spline algorithm.
- Train the medical transformer network through real training and simulated data.
- Test the trained model, and obtain the final segment results.

### Image data augmentation based on the deformable model

The image data strategy combines the statistical shape model (SSM) and thin plate spline to generate new training images. The statistical shape model is a commonly used statistical method for feature positioning. We first build a statistical shape model based on the contour shape of the target organ in training data, which is then used to generate the simulated shape of the target organ. Given N sets of two-dimensional or three-dimensional training samples by their shapes ${\{{s}_{i}\}}_{i=1}^{N}$. The shape model can be represented by:

1 $$s=\overline{{s }_{0}}+\mathbf{E}\mathbf{b}$$

where $\mathbf{b}=\{{b}_{1},{b}_{2},\dots ,{b}_{k}\}$ is the shape parameters, including scale and rotation parameters. By changing the value of **b**, we can generate any simulated shapes from this model. Typically, the range of **b** should lie in a hyperrectangle $\mathbf{b}\le \alpha \sqrt{{\varvec{\uplambda}}}$ with $\alpha \in [-1.5,1.5]$. The simulated shape generated by the algorithm is more in line with the distribution of the actual human organs.

Then, we generate the texture of each simulated shape from training data with the 2D thin plate spline algorithm. The 2D thin plate spline function can be specified as follows:

2 $$f(x,y)=\left(\begin{array}{c}{a}_{1x}\\ {a}_{1y}\end{array}\right)+\left(\begin{array}{c}{a}_{2x}\\ {a}_{2y}\end{array}\right)x+\left(\begin{array}{c}{a}_{3x}\\ {a}_{3y}\end{array}\right)y+\sum_{i=1}^{n}\left(\begin{array}{c}{w}_{ix}\\ {w}_{iy}\end{array}\right)U(\left|{p}_{i}-(x,y)\right|)$$

The first part is an affine transformation representing the behavior of $f(x,y)$ at infinity. The second part is the weighted sum of root function $U(r)={r}^{2}log({r}^{2})$. According to the function $f(x,y)$, any points $t=(x,y)$ in the simulated image can be transformed into the points ${t}^{\mathrm{^{\prime}}}=({x}^{\mathrm{^{\prime}}},{y}^{\mathrm{^{\prime}}})$ in the real image, then insert gray values of point ${t}^{\mathrm{^{\prime}}}$ in the real image into the point $t$ in the simulated image.

We can augment the training data with realistic simulated images by combining shape generation and texture interpolation methods.

### Gated axial-attention

Due to the inherent inductive preference of convolutional structures, it lacks the ability to model remote dependencies in images. Transformer constructs use self-attention mechanisms to encode long-distance dependencies and learn highly expressive features. We add the transformer structure into the network to improve the ability of network feature expression and location.

**Fig. 2:**
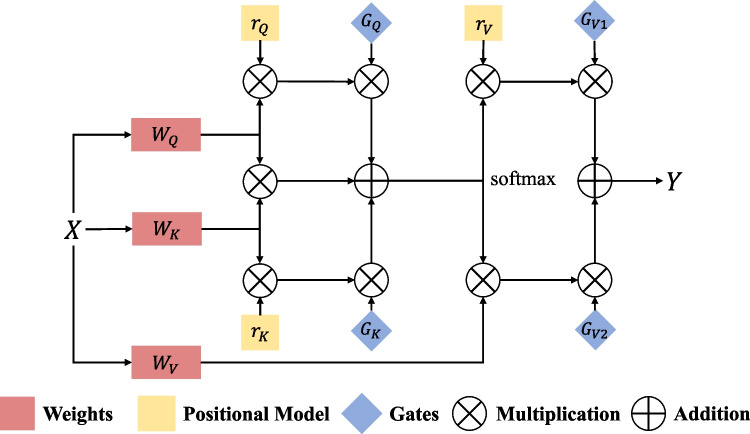
Main structure of the gated axial attention mechanism network

**Fig. 3:**
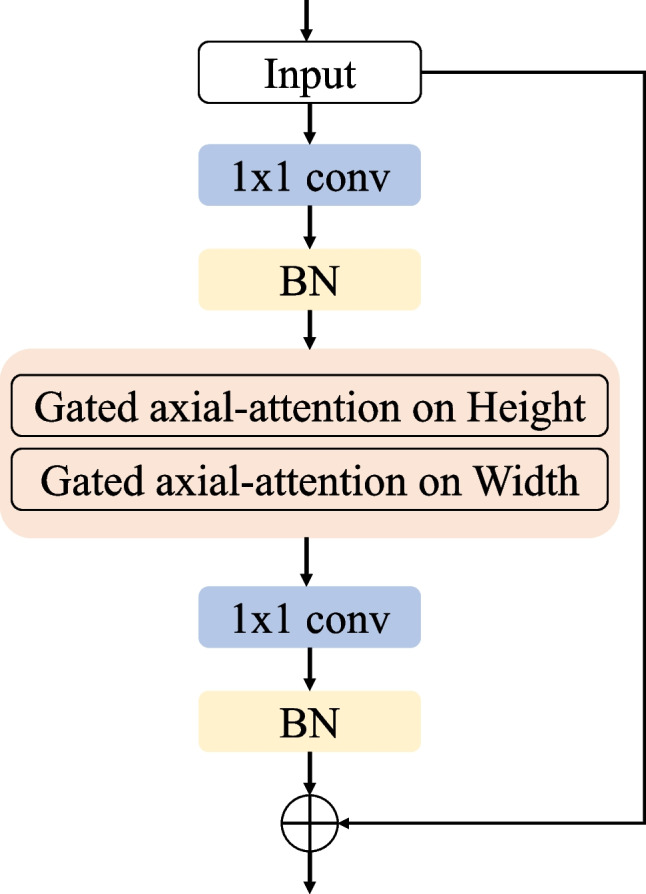
The structure of encoders

We adopt an axial attention-based method to extend the existing structure. This additional positional bias in Query, Key, and Value captures remote interactions with precise positional information. For any given input feature $x$, the axial self-attention mechanism with relative position encoding and width axis can be written as:

3 $${y}_{ij}=\sum_{\omega =1}^{W}softmax({q}_{ij}^{T}{k}_{iw}+{q}_{ij}^{T}{r}_{iw}^{q}+{k}_{iw}^{T}{r}_{iw}^{k})({v}_{iw}+{r}_{iw}^{v})$$

where, ${r}^{q},{r}^{k},{r}^{v}\in {\mathbb{R}}^{W\times W}$ are axial attentional models in the width direction. Formula (3) describes the axial attention applied along the tensor width axis. A similar formula is also used to apply axial attention along the height axis. Axial attention can compute non-local contexts with good computational efficiency, encode positional biases into mechanisms, and encode remote interactions in input feature graphs. However, it is difficult to learn in experiments with small-scale data sets that often occur in medical image segmentation, so it is not always accurate when encoding remote interactions. Adding relative positions to their respective keys, queries, and values can lead to performance degradation if the relative positions learned are not encoded accurately enough. Therefore, we use an improved axial block, shown in Figs. 2 and 3, which can control the effect of position deviation on non-local context encoding. With the proposed modification, the self-attention mechanism applied to the width axis can be formally written as:

4 $${y}_{ij}=\sum_{\omega =1}^{W}softmax({q}_{ij}^{T}{k}_{iw}+{G}_{Q}{q}_{ij}^{T}{r}_{iw}^{q}+{G}_{K}{k}_{iw}^{T}{r}_{iw}^{k})({G}_{{V}_{1}}{v}_{iw}+{G}_{{V}_{2}}{r}_{iw}^{v})$$

Formula (4) is very close to Formula (3) but adds the gating mechanism. ${G}_{Q},{G}_{\mathrm{K}},{G}_{{V}_{1}},{G}_{{V}_{2}}\in {\mathbb{R}}$ are learnable parameters that together form the gating mechanism for controlling the effect of learning relative position encoding on non-local context encoding. Generally, if a relative position code is accurately learned, the gating mechanism will assign a higher weight than those not.

### Main structure of the medical transformer network

The structure of the encoder is shown in Fig. 3 below. Each decoder has typical convolution layers using a 1 × 1 convolution kernel, a BN layer, Gated axial-attention layers, and BN layers. The decoder part has one convolution layer, one up-sampling layer, and one ReLU activation. Between each pair of encoder and decoder, there is a skip connection. In the expanded path, each decoder step includes an up-sampling operation of the feature map. After up-sampling, the number of feature channels will be reduced by half, then jump to the corresponding feature map in the contraction path. Then convolution operations are used with three convolution layers, all using BN and ReLU activation functions. It should be emphasized that an additional 1 × 1 convolution layer is connected after feature mapping in the training process.

Due to the small sample size of medical image data, it is difficult to train the transformer network for medical images effectively. We divide our network into two branches. In the first branch, 1 × 1 convolution is performed on the whole image, and then a two-layer encoder and a two-layer decoder are used and skip connections are made. In the second branch, the original image is evenly divided into 16 small images using the multi-layer encoder and decoder method, and resampling is carried out. The feature is resampled and weighted with the feature image in the first branch, and the final segmented image is obtained after passing through a 1 × 1 convolution layer. This strategy can improve the segmentation performance of the network and pay attention to the global high-level information. At the same time, the local branch can pay attention to finer details so that the segmentation can be more accurate.

The model adopts the binary cross-entropy loss, and its specific definition is as follows:

5 $${\text{Loss}}\left(\text{P, }\widehat{\text{P}}\right)\text{ = }-\frac{1}{{\text{N}}}\sum_{{\text{i}}= \text{1} }^{\text{N}}{p}_{i}\bullet \mathrm{log}\left(\widehat{{p}_{i}}\right)+(1-{p}_{i})\mathrm{log}(1-\widehat{{p}_{i}})$$

where ${p}_{i}$ is the true label of the pixel, and $\widehat{{p}_{i}}$ is the predicted probability of the pixel for all N points.

## Results

### Datasets

We used the National Cancer Institute (NCI) Cancer Imaging Program and the Prostate Magnetic Resonance Imaging Public Data released by the International Society of Biomedical Imaging (ISBI). The data published by NCI-ISBI contains 80 sets of 3D data fields, including 60 sets of training data sets, 10 sets of validation data sets and 10 sets of test data sets. Half of the images are obtained by a magnetic resonance machine with a magnetic field strength of 1.5 T, and the other half are acquired by a magnetic resonance machine with a magnetic field strength of 3 T. Because one data set does not match the gold standard in the training data, the correct segmentation accuracy cannot be obtained, so it is excluded from the experiment. We sliced the three-dimensional data in the transverse direction. Then we got 10,155 two-dimensional training images, 1825 verification images, and 1830 test images.

The second dataset is echocardiography. The dataset contains 480 transverse images, and the image size is 800 × 600. Two radiologists labeled the region of the left ventricular valve. The experiment randomly selected 360 images as training sets and the remaining 120 as test sets.

The third dataset is the tongue image from the web (https://github.com/BioHit/TongeImageDataset). These data were collected by the professional tongue diagnostic instrument. The public data contains 300 sets of tongue pictures with labels. To enhance the complexity of the data, we recruited 100 volunteers from Longhua Hospital Affiliated to Shanghai University of Traditional Chinese Medicine and collected their tongue images with a digital camera. Thus, we collected a total of 400 images. To verify the model's ability to handle noisy data, we added Gaussian noise ($\mu =0, {\sigma }^{2}=0.02$) to 20% of the images. Then, the data were randomly divided into 320 training and 80 test datasets. It's important to emphasize that observing the tongue is a unique part of Traditional Chinese Medicine (TCM)[30]. Segmenting the tongue from the image can provide a solid foundation for subsequent quantitative analysis.

### Metric

To verify the performance of the proposed network, we use Dice’s similarity coefficient (DSC) to measure the segmentation algorithm, which is defined explicitly as Formula (6), (7) and (8), where **X** and **Y** represent the algorithm segmentation result and the gold standard, respectively.

6 $$\mathrm{DSC}=\frac{2\left|\mathbf{X}\cap \mathbf{Y}\right|}{\left|\mathbf{X}\right|+\left|\mathbf{Y}\right|}$$

7 $$\mathrm{IoU}=\frac{\left|\mathbf{X}\cap \mathbf{Y}\right|}{\left|\mathbf{X}\cup \mathbf{Y}\right|}$$

8 $$\mathrm{Recall}=\frac{\left|\mathbf{X}\cap \mathbf{Y}\right|}{\left|\mathbf{Y}\right|}$$

We choose to segment three medical images to demonstrate the effectiveness of our method, including prostate MR image, heart US image, and tongue images. We augment the training set by 30% for each task with our proposed algorithm.

### Performance of the algorithm

**Fig. 4:**
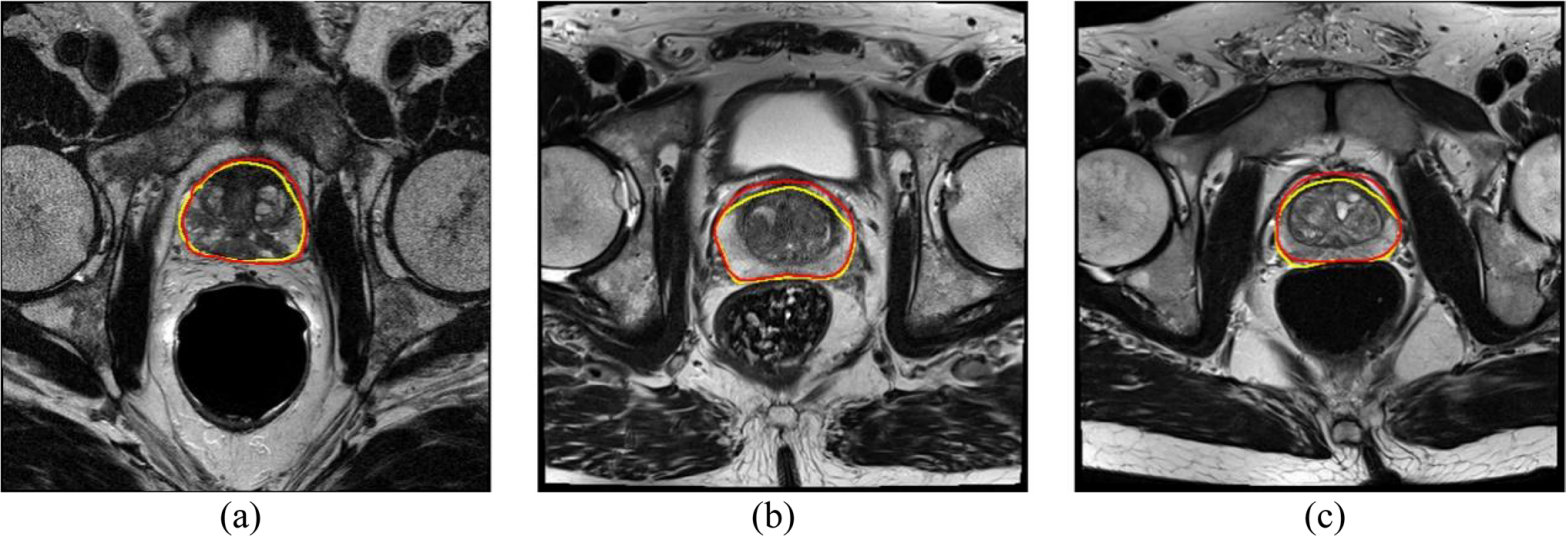
Results of prostate image segmentation, where the yellow contours are the gold standard and the red contours are the algorithm segmentation result

Compare with other prostate segmentation methods: CNN based method [31], Super Voxel-based method [32], U-net [18], R2U-net [33], Att U-net [34] and U-net +  + [21]. U-net, R2U-net, Att U-net and U-net +  + are reproduced through the article. The hyper-parameters of the above models are basically the same, such as the learning-rate is set to 1e-4, the epoch is set to 25, the batch-size is 4, all use Adam with Momentum optimizer. The accuracy results of other methods are derived from the corresponding papers. The comparison of the segmentation accuracy is shown in Table 1. The proposed method obtains a competitive result among the fully automatic segmentation algorithms. Figure 4 shows the segmentation results.

**Table 1:** Segmentation results of different algorithms on prostate MR image test datasets

Method	DSC	IoU	Recall
CNN + CRF-RNN	78.20%	-	-
Super voxel	88.23%	-	-
U-net	82.02%	69.52%	76.13%
R2U-net	86.49%	76.20%	84.60%
Att U-net	88.91%	80.03%	**87.04%**
U-net + +	88.51%	79.38%	85.18%
Our method	**89.97%**	**81.78%**	86.36%

**Fig. 5:**
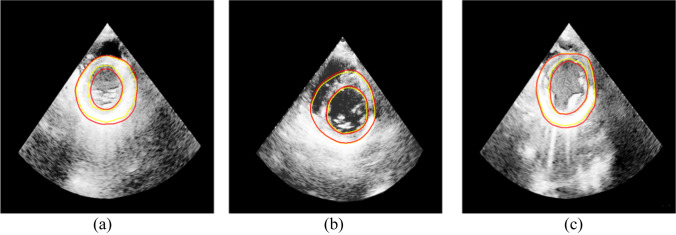
The segmentation result, where the golden contours represent the real annotation result, and the red contours are the segmented image

Since the ultrasound images suffer from low contrast and poor imaging quality, we performed image preprocessing, including mask operation and gray level equalization. We compare our method with other segmentation methods( U-net [18], R2U-net [33], Att U-net [34] and U-net +  + [21]). The above networks are reproduced by their original papers. The segmentation accuracy is shown in Table 2. The DSC of our method is 91.90%. Figure 5 shows the transverse plane segmentation results of echocardiography.

**Table 2:** Segmentation results of different algorithms on heart US image

Method	DSC	IoU	Recall
U-net	87.34%	77.55%	87.18%
R2U-net	90.04%	81.88%	81.94%
Att U-net	90.25%	82.23%	86.18%
U-net + +	90.36%	82.42%	90.02%
Our method	**91.90%**	**85.01%**	**90.18%**

**Fig. 6:**
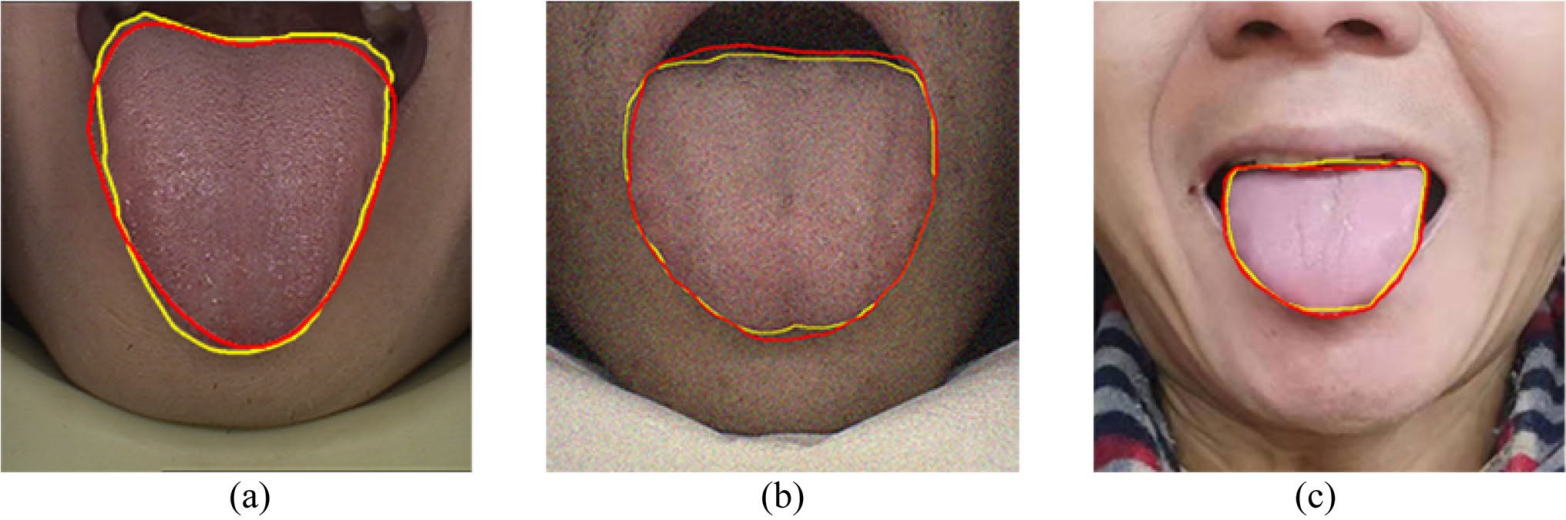
The segmentation result of the tongue image, where the golden contours represent the real annotation result, and the red contours are the segmented image. (**a**) Original image of the public dataset. (**b**) Images with Gaussian noise. (**c**) Images captured by the digital camera

No preprocessing was done for the tongue image dataset except for scale normalization. We compare our method with other tongue image segmentation methods(U-net [18], R2U-net [33], Att U-net [34] and U-net +  + [21]). The above networks are reproduced by their original papers. The segmentation accuracy is shown in Table 3. Figure 6 shows the segmentation results of tongue images.

**Table 3:** Segmentation results of different algorithms on tongue color image

Method	DSC	IoU	Sen
U-net	89.69%	81.31%	84.83%
R2U-net	87.37%	77.96%	84.59%
Att U-net	93.19%	88.65%	93.53%
U-net + +	93.77%	88.26%	**96.50%**
Our method	**94.25%**	**89.12%**	93.28%

## Discussion

In recent years, deep learning has achieved outstanding results in many fields. However, the available training data for medical images are often scarce. We combined the deformable model with the medical transformer network to achieve medical image segmentation with a small amount of data. Theoretically, this method can effectively increase the information flow of the network so that less training data can be used to achieve medical image segmentation.

We used the three types of medical images to verify the performance of the segmentation algorithm based on our method. The steps are as follows: First, we preprocessed the training image, including uniform image size and grayscale normalization. Second, we used the statistic shape model and 3D thin plate spline to achieve the purpose of data augmentation. Third, we constructed the medical transformer network structure to segment three types of medical images. The test results show that the segmentation algorithm proposed in this paper achieved a DSC of 89.97%, 91.90%, and 94.25% on the prostate MR images, heart US images and tongue color images, respectively.

Of course, the algorithm still has the following limitations, such as the medical transformer used in this paper is still based on the 2D images, and some of the 3D spatial information of the image is lost. The main reason for the above shortcomings lies in too few labeled training data (there are only 59 training data sets).

Our future work will focus on the following directions. First, the medical transformer network can be applied to other medical image segmentation tasks, such as cardiac MR images, etc. Second, the network can be extended to 3D space, fully using 3D spatial information. Third, the network can be further improved to build a deep neural network with better performance.

## Conclusion

In this paper, we combined the deformable model and medical transformer network to achieve image segmentation. The proposed method can alleviate the problem of fewer labeled medical images. The method was tested on three types of medical images, including prostate MR image, heart US image, and tongue color images. Our method achieved higher accuracy than the common model used in medical image segmentation.
